# Into the wild: How exposure to wild or domesticated fungi shapes immune responses in mice

**DOI:** 10.1371/journal.ppat.1010841

**Published:** 2022-10-13

**Authors:** Ying-Han Chen, Ken Cadwell

**Affiliations:** 1 Kimmel Center for Biology and Medicine at the Skirball Institute, New York University Grossman School of Medicine, New York, New York, United States of America; 2 Department of Microbiology, New York University Grossman School of Medicine, New York, New York, United States of America; 3 Division of Gastroenterology and Hepatology, Department of Medicine, New York University Grossman School of Medicine, New York, New York, United States of America; University of Maryland, Baltimore, UNITED STATES

## What is the gut mycobiota and why should we care?

Throughout our lives, humans are exposed to diverse microbes that impact the development and function of our immune system. As an immense and absorptive mucosal surface exposed to the external environment, the gastrointestinal (GI) tract is a major interface in which these immunogenic interactions occur. Human subject studies have linked disruption of the intestinal bacterial community to a variety of disease conditions, and experimental models have helped us interpret these findings by elucidating mechanisms by which bacteria evoke pro- or anti-inflammatory responses through host cells and receptors. More recent studies are beginning to define the impact of nonbacterial agents in the gut such as fungi.

The collection of fungi present in the GI tract is referred to as the gut mycobiota and can be considered part of the larger community of microbiota and meiofauna inhabitants consisting of bacteria, archaea, viruses, protozoa, and helminths. Although relatively low in abundance compared with bacteria, fungi are ubiquitous colonizers with a large role in the developmental succession of the infant microbiota [1]. Genera of fungi in the human gut include *Candida*, *Saccharomyces*, *Aspergillus*, and *Malassezia*. However, at least some of the fungi routinely detected in the human gut are transient passengers ingested with food [2]. Stable colonization may require adaptation to the host GI tract, such as evasion of antibody responses and the ability to switch between yeast and hyphal cell morphology [3–5].

Early-life exposure to fungi is associated with reduced prevalence of wheezing and asthma later in childhood [6]. Also, alterations in the abundance of fungi and colonization by particular strains, such as those that produce toxins at high levels, are linked with susceptibility to immune-mediated disorders, most notably inflammatory bowel disease (IBD) [7–10]. Medical interventions that disrupt the balanced coexistence of intestinal fungi and bacteria, such as allogeneic hematopoietic cell transplantation, can lead to disastrous health consequences for the host [11]. These observations reveal the existence of a consequential symbiotic relationship with a community of intestinal fungi.

## What local responses are triggered during intestinal colonization by fungi?

Numerous mechanisms restrict the ability of fungi to establish invasive infections or diseases in the GI tract. In addition to Toll-like receptors (TLRs) and nucleotide oligomerization domain (NOD)-like receptors (NLRs) well known in the innate immunity field, pattern-recognition C-type lectin receptors (CLRs) play a central role in fungal recognition. CLRs Dectin-1, Dectin-2, and Mincle activate Spleen tyrosine kinase (Syk) upon binding carbohydrate polymers of the fungal cell wall such as β-glucan, mannans, and chitin, trigger signaling pathways leading to cytokine production, phagocytosis, and other effector functions. In the gut, CX3CR1+ mononuclear phagocytes (MNPs) highly express CLRs and downstream signaling mediators and, following recognition of fungi, migrate to the draining lymph nodes to induce the differentiation of naïve T cells into T helper 17 (Th17) cells and production of antifungal antibodies [12]. Some fungi produce gliotoxin and other metabolic by-products that are toxic to host cells. Macrophages in the colon extend balloon-like protrusions into the lumen that absorb fluids containing these mycotoxins to prevent their hazardous uptake by the epithelium [13]. These processes act in concert to limit fungal overgrowth, avoid mycobiota dysbiosis, and prevent inflammatory damage.

## Does colonization by intestinal fungi contribute to the normal development of the immune system?

Laboratory mice are broadly used for studying how the microbiota contributes to the maturation and function of the immune system. However, there is growing evidence that the limited microbial exposure experienced by laboratory mice kept in ultra-hygienic specific pathogen-free (SPF) facilities leads to a less mature immune system when compared with free-living mammals such as humans. Laboratory mice exposed to feral or pet store mice, also referred to as “dirty” mice, better recapitulate observations made in humans in models of infectious disease, colorectal cancer, and pharmaceutical interventions [14–17]. In these experiments, immune maturation is associated with exposure to disease-causing transmissible agents (i.e., pathogens), especially viruses and parasites. Do fungi contribute to maturation of the immune system?

**Fig 1:**
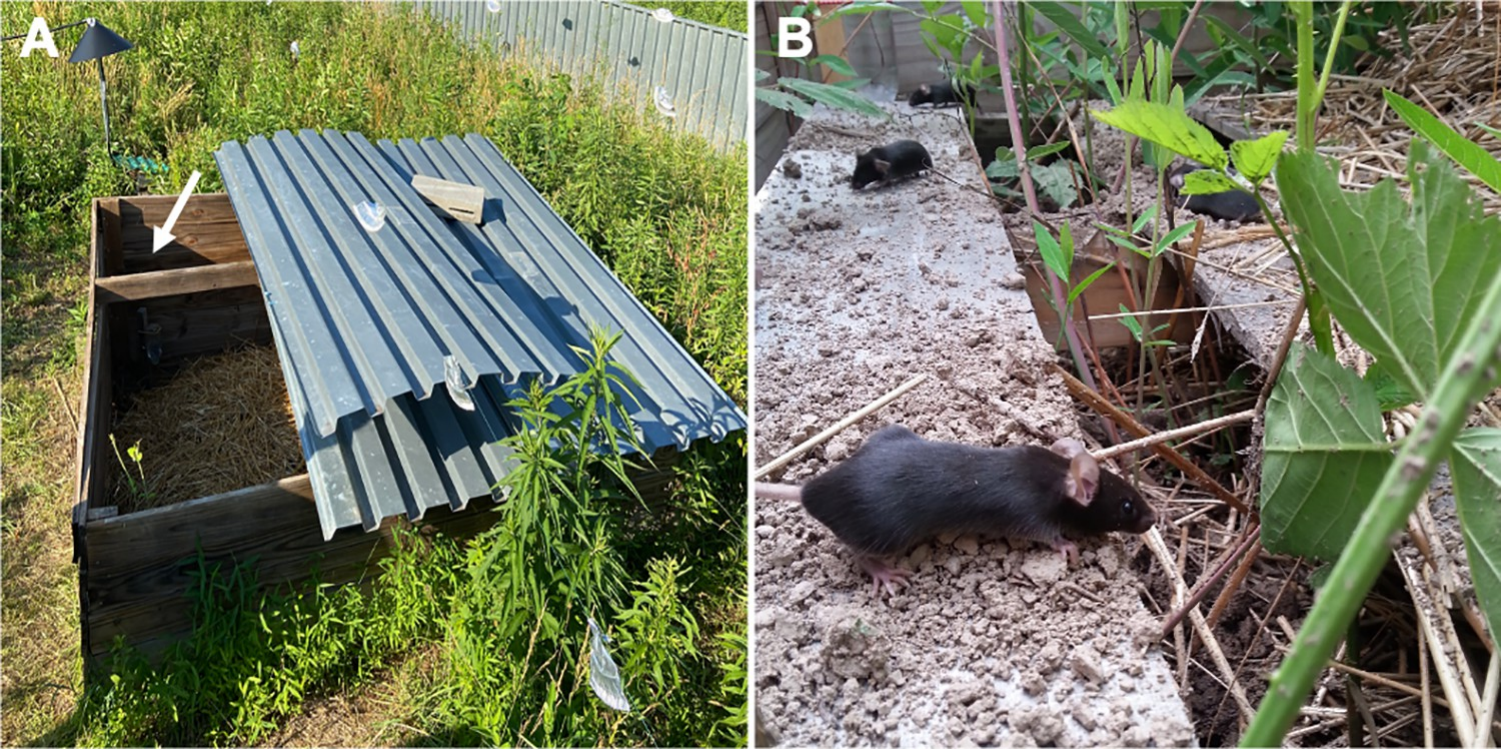
Free-range laboratory mice. (**A**) A close-up view of a wedge in the Stony Ford outdoor enclosure facility. Individual wedges contain 2 watering stations and a feeding station (yellow star) supplied with the same mouse chow that laboratory mice receive in the institutional vivarium. (**B**) During the process of rewilding, adult C57BL/6J mice raised in a specific pathogen-free (SPF) facility are introduced into the enclosure where they are exposed to fungi in the environment as they explore their surroundings. *Photo courtesy of Christina B*. *Hansen and Andrea Graham*, *Princeton University*.

Laboratory mice that experience a seminatural environment through release into an outdoor enclosure facility, a process referred to as “rewilding,” display >100-fold increases in fungal burden [18]. In this outdoor enclosure, the zinced iron walls with electric fencing excludes predators and rodents harboring pathogens, while allowing exposure to natural soil, vegetation, insects, and weather (Fig 1). As such, rewilded laboratory mice are exposed to environmental microbes (i.e., microbes acquired from the environment rather than through horizontal transfer from another rodent) but remain seronegative for pathogens excluded from SPF facilities. The fungal mycobiota of rewilded mice resembles wild mice [16] and is characterized by increased diversity and acquisition of Ascomycota phylum members, especially *Aspergillus* species. In addition to exhibiting hallmarks of increased lymphocyte differentiation and cytokine production, rewilded mice display a striking expansion of granulocytes, a group of myeloid lineage white blood cells such as neutrophils [18,19]. Granulocyte numbers in the blood correlate with fungal burden, and transferring fungi isolated from the feces of rewilded mice into laboratory mice reproduces the increase in peripheral granulocytes. Similarly, colonizing the intestines of laboratory mice with *Candida albicans* leads to an increase in circulating granulocytes, and reducing fungal burden with the antifungal drug fluconazole leads to a corresponding decrease in granulocytes. Hence, fungi in the natural environment contribute to the composition of circulating immune cells.

It is possible that rewilded mice represent an intermediary condition of microbial exposure occupying a state in between SPF and feral mice. Rather than a binary status of “clean versus dirty” or “naïve versus exposed,” immune maturation induced by microbes likely exists in a continuum. Humans display interindividual differences in their history of infections. Incorporating mouse models with different degrees of microbial exposure including fungi may help capture some of this diversity.

## In what other ways does intestinal colonization by fungi affect physiology?

Human Th17 cells reactive to gut-resident *C*. *albicans* are cross-reactive with airborne fungi [20], and in mice, this Th17 response protects against invasive bacterial and fungal infections [21,22]. However, *C*. *albicans* strains recovered from the intestines of IBD patients display high cytotoxicity and induce greater production of the inflammatory cytokine IL-1β from macrophages compared with non-IBD isolates [7], highlighting the fine line between beneficial and adverse immune reactions to fungal colonization. Adverse consequences of an altered gut mycobiota are not limited to the GI tract. In mouse models of allergic airway inflammation, administration of antimicrobials that cause overgrowth of intestinal fungi can exacerbate disease through multiple mechanisms, including increasing prostaglandin E2 (PGE2) levels that alter the polarity of lung macrophages and local activation of CX3CR1+ MNPs that promote long-distance eosinophil infiltration [23,24]. Alteration of gut mycobiota composition and burden are also linked to alcoholic hepatitis through the translocation of β-glucan into systemic circulation [25]. Another study found a positive correlation between alcoholic hepatitis and fecal candidalysin, an exotoxin secreted by *C*. *albicans* [26]. Lastly, systemic IL-17A induced by mucosal fungi acts on neurons to regulate social behavior in mice, demonstrating how the gut mycobiota can contribute to the gut–brain axis [27].

## Can we improve disease models by incorporating intestinal fungi?

How the gut mycobiota influences the host may be useful to consider when attempting to model disease events in laboratory mice because intestinal fungi are generally low in abundance or undetectable by culturing methods in mice bred in SPF facilities. For example, blood neutrophil levels are lower in laboratory mice than in humans. As a major phagocyte population and first line defenders during infections, this discrepancy may confound attempts to fully utilize the mouse model to study immunity. Recent evidence highlights a previously unappreciated heterogeneity of neutrophils, especially mature neutrophils in the periphery, which may be key for their function during chronic conditions, such as cardiovascular inflammation [28]. The diversity of neutrophils was revealed by single-cell RNA sequencing analysis, which identified an expanded subset enriched in the expression of interferon (IFN)-stimulated genes during bacterial infection [29]. In addition to increasing the number of circulating neutrophils, does GI colonization by fungi impact neutrophil maturation and function?

While colonizing laboratory mice with a genetically tractable model fungus represents an accessible reductionist approach to address these types of questions, it would be highly informative to fully characterize rewilded mice, which display alterations in abundance and composition of both the gut mycobiota and bacterial microbiota. Other experimental systems add another level of complexity by introducing viruses, which have broad effects on the mucosal immune system [30,31]. Knowledge gained from comparing different gradations of microbial exposure will better define the relative contribution of intestinal fungi to immunity and may help us improve the mouse model for investigating the biology of free-living mammals.
